# Artificial Intelligence-Assisted Virtual Reality for Reducing Anxiety in Pediatric Endoscopy

**DOI:** 10.3390/jcm14041344

**Published:** 2025-02-18

**Authors:** Mehmet Bulduk, Veysel Can, Emre Aktaş, Belkıs İpekçi, Bahattin Bulduk, İbrahim Nas

**Affiliations:** 1 Faculty of Health Sciences, Department of Nursing, Van Yüzüncü Yıl University, 65000 Van, Turkey; veyselcan@yyu.edu.tr (V.C.); bahattinbulduk@yyu.edu.tr (B.B.); ibrahimnas@yyu.edu.tr (İ.N.); 2 Van Regional Training and Research Hospital, 65000 Van, Turkey; emre_aktas13@hotmail.com (E.A.); belkisipekci@hotmail.com (B.İ.)

**Keywords:** artificial intelligence, virtual reality, endoscopy, gastrointestinal, anxiety, preoperative, non-pharmacological therapy

## Abstract

**Background/Objectives:** This study aimed to evaluate the effects of artificial intelligence (AI)-assisted virtual reality (VR) applications on preoperative anxiety levels and vital signs in children undergoing endoscopy. **Methods:** A randomized controlled trial design was employed, including a total of 80 children aged 8–17 years (40 in the intervention group and 40 in the control group). Children in the intervention group were exposed to VR applications featuring space and underwater themes, while the control group received standard procedures. Anxiety levels were assessed using the “State-Trait Anxiety Inventory for Children (STAIC)”, and vital signs were evaluated through measurements of systolic and diastolic blood pressure, heart rate, temperature, and SpO_2_. **Results:** VR applications significantly reduced anxiety scores in the intervention group (36.3 ± 1.9), while no significant changes were observed in the control group (45.4 ± 2.74) (*p* < 0.001). Regarding vital signs, more favorable outcomes were observed in the intervention group. Systolic blood pressure was measured as 89 ± 6.7 mmHg in the intervention group and 96.5 ± 10.5 mmHg in the control group (*p* < 0.001). Diastolic blood pressure was 60.8 ± 4.7 mmHg in the intervention group and 63.8 ± 6 mmHg in the control group (*p* < 0.05). Heart rate was recorded as 88.7 ± 10.1 bpm in the intervention group and 94.5 ± 14.8 bpm in the control group (*p* < 0.05). SpO_2_ levels were 98 ± 1 in the intervention group and 96.2 ± 1.3 in the control group (*p* < 0.001). **Conclusions:** AI-assisted VR applications emerge as an effective non-pharmacological method for reducing preoperative anxiety and promoting physiological stability in children. This approach holds the potential to enhance pediatric experiences during invasive procedures such as endoscopy.

## 1. Introduction

Medical procedures, particularly invasive interventions, can lead to heightened preoperative anxiety levels in children due to their cognitive limitations and dependency [1]. Even a simple and well-planned surgery can be perceived as a threat to the child’s physical integrity, potentially resulting in severe levels of anxiety [2]. Preoperative anxiety may manifest through symptoms such as palpitations, rapid heartbeat, irregular heart rhythms, nausea, shortness of breath, and sleep disturbances [3]. Such intense anxiety can negatively affect children’s physical and psychological health, delay recovery and rehabilitation processes, and reduce their cooperation in self-care activities [4,5,6]. Additionally, it has been noted that patients experiencing preoperative anxiety are three times more likely to develop postoperative anxiety [7].

Non-pharmacological interventions offer a significant alternative for reducing anxiety in children and making the preoperative process more manageable. Among these interventions, distraction-based techniques are increasingly preferred to reduce or eliminate the need for sedative applications [8,9]. Distraction methods include educational programs [10], web-based mobile health interventions [11], clown interventions [12], interactive games [6], and virtual reality applications [13]. VR has gained attention as an effective non-pharmacological method for anxiety reduction, emerging as a complex yet powerful tool during medical procedures [14,15,16]. Studies have demonstrated that preoperative VR technologies reduce children’s anxiety levels and enhance their cooperation [17,18]. However, adverse responses to surgical procedures vary among children due to individual differences, which can influence the effectiveness of these interventions [19].

Artificial intelligence focuses on creating content that captures children’s attention and provides a soothing experience. In this study, space and underwater themes were chosen because they represent unknown and largely unexplored realms for both children and adults [20,21,22]. Similarly, endoscopy is a medical procedure that carries an element of uncertainty for children. However, AI-generated vibrant VR visuals transform these unfamiliar environments from potentially intimidating experiences into engaging explorations that stimulate curiosity and discovery.

This study demonstrates that AI can facilitate the rapid and cost-effective development of VR content, which can be tailored to each child’s individual interests. Due to its flexible nature, AI-generated VR content can be customized not only to accommodate children’s personal preferences but also to align with specific medical procedures performed in clinical settings. As research in this area progresses, VR applications may be further adapted based on children’s age, interests, and anxiety levels, as well as the characteristics of the medical procedures they undergo.

Accordingly, this study aims to evaluate the impact of AI-assisted VR applications on preoperative anxiety levels in children undergoing endoscopy and to introduce a novel perspective for clinical practice.

## 2. Materials and Methods

### 2.1. Study Design

This study is a randomized controlled intervention designed to evaluate the effects of virtual reality (VR) applications on pre-endoscopy anxiety levels and vital signs in children aged 8–17 years. The effectiveness of the VR application was examined by comparing the intervention and control groups. Children in the intervention group were shown AI-designed virtual reality visuals with space and underwater themes, developed based on expert opinions, through VR headsets. In contrast, children in the control group were subjected to standard procedures.

### 2.2. Study Location and Time

This study was conducted between August 2024 and December 2024 in a tertiary hospital located in the eastern region of Turkey. During this period, all patients admitted to the pediatric gastroenterology clinic were evaluated and selected based on eligibility criteria.

### 2.3. Participants and Sample

The 8–17 age range was selected because it aligns with the applicability of the State-Trait Anxiety Inventory for Children (STAIC), ensuring that the scale could be effectively administered and reliably measure anxiety levels within this population [23,24]. The study included children aged 8–17 years who were admitted to the pediatric gastroenterology clinic for planned endoscopy due to intestinal issues (chronic diarrhea, chronic constipation, Crohn’s disease), esophageal problems (esophageal reflux, esophageal stricture), gastric conditions (gastritis, stomach ulcers), or general gastrointestinal concerns (hematemesis, hemoptysis, foreign body ingestion, or unexplained gastrointestinal complaints). Throughout the study period, a total of 182 children who presented to the clinic were evaluated, and 80 children (40 in the intervention group and 40 in the control group) who met the inclusion criteria were enrolled. The study was concluded once the required sample size was reached. Before the procedure, the children were admitted to the pediatric clinics and written and verbal informed consent was obtained from both the children and their families after providing detailed information about the study.

#### 2.3.1. Inclusion Criteria

- Children aged 8 to 17 years.
- Scheduled to undergo an endoscopy procedure.
- Able to provide assent, and their parents or legal guardians provide written informed consent.
- No prior exposure to virtual reality applications.
- Willing to participate in the study.

#### 2.3.2. Exclusion Criteria

- Presence of a chronic medical condition affecting cardiovascular or respiratory systems.
- Diagnosed with a severe psychiatric disorder.
- Known visual or auditory impairments that could prevent participation in a virtual reality session.
- History of seizures or epilepsy.
- Unwillingness to participate or inability to comply with study procedures.

#### 2.3.3. Randomization

**Figure 1:**
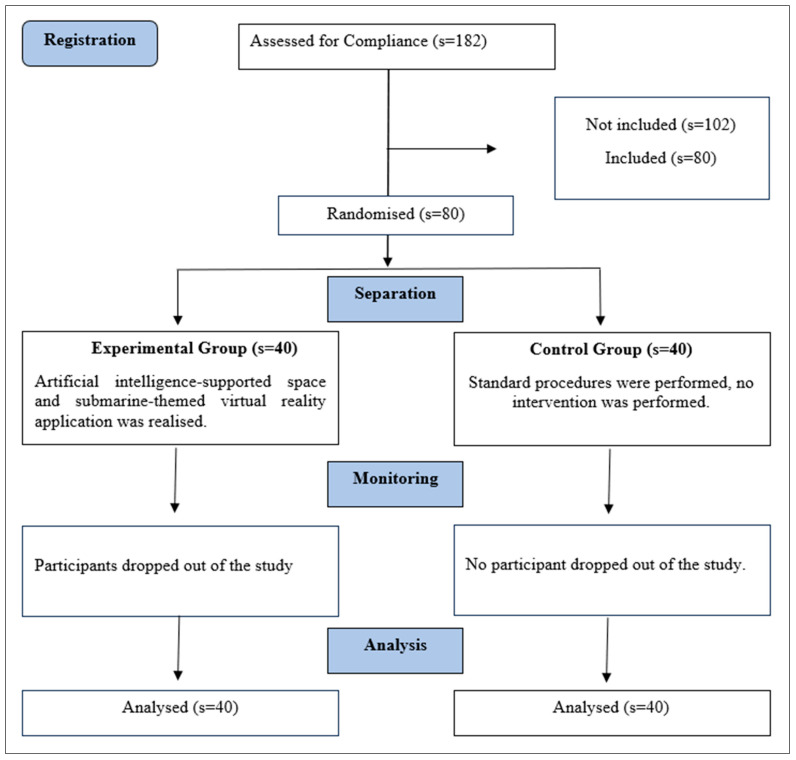
CONSORT Flow Diagram.

Participants were randomly and equally assigned to the intervention and control groups. A simple random selection method was used for the randomization process. Endoscopy procedures were performed one day per week. Patients scheduled for the procedure on this day were admitted to the pediatric clinic one day prior and taken to the preoperative waiting room on the day of the endoscopy. Each patient in the pre-op room was assigned a random code, and the interventions were applied based on these codes. The codes were generated using computer software, and group information was kept confidential by the researcher. Both the intervention and control procedures were implemented by the researcher (Figure 1).

#### 2.3.4. Blinding of Evaluators

To minimize the risk of bias during data collection, evaluator blinding was implemented. The nurse responsible for measuring vital signs and administering the questionnaires was unaware of the participants’ group assignments. The nurse only accessed participant codes, and all measurements were performed following the same standardized procedures. Both groups of children underwent identical measurement and evaluation protocols. However, since the intervention created a visual distinction, strict adherence to standard protocols was ensured to minimize potential biases.

#### 2.3.5. Intervention

Children in the experimental group were presented with AI-assisted VR content featuring space and underwater-themed visuals, delivered via a virtual reality headset for a duration of 7 min.

#### 2.3.6. AI Utilization in Visual Design

**Figure 2:**
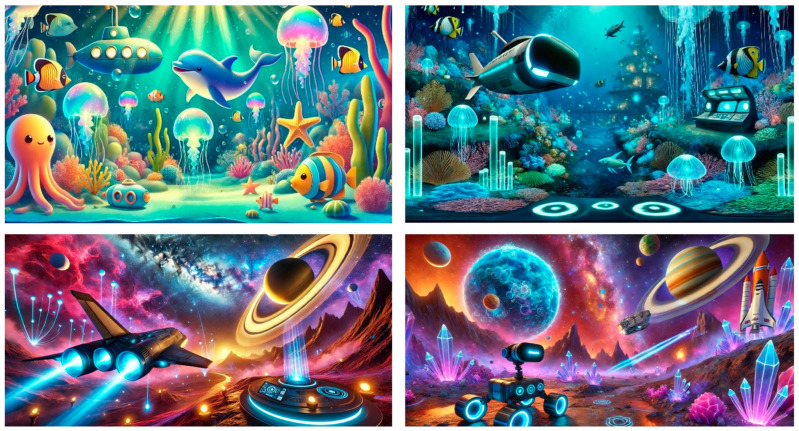
Screenshots from the video created using artificial intelligence programs.

Artificial intelligence was employed using computer vision, deep learning, and content optimization algorithms to tailor visual content according to the participants’ age groups. Figure 2 illustrates the AI-generated visuals used in the intervention.

- For the 8–11 age group, more vibrant colors and simplified objects were incorporated. The visuals were designed to be dynamic and engaging, ensuring that children with shorter attention spans could remain focused.
- For the 12–17 age group, the visuals were designed to be more detailed and realistic, featuring smoother transitions and an emphasis on exploration to align with the cognitive development of older participants.

#### 2.3.7. Calming Music and Visual-Auditory Integration

The music selection was AI-assisted and customized according to age groups, incorporating classical music, nature sounds, and instrumental melodies to create a low-tempo, soothing auditory environment.

- For the 8–11 age group, more melodic and rhythmic music was chosen.
- For the 12–17 age group, slower, minimal instrumental compositions were used.

AI evaluated the frequency spectrum, rhythm, and tonal components of the selected tracks to ensure a harmonized sensory experience. The integration of visual and auditory stimuli was strategically designed to complement each other, rather than functioning as isolated elements.

#### 2.3.8. Assessment

Children’s anxiety levels were assessed using the validated and reliable Children’s State Anxiety Scale (CSAS), while vital signs (systolic and diastolic blood pressure, heart rate, temperature, and SpO_2_) were measured and recorded.

#### 2.3.9. Data Collection Tools

The following tools were used to collect data in this study:

- Patient Identification Form: Developed by the researchers to determine the demographic and clinical characteristics of the participants.
- State-Trait Anxiety Inventory for Children (STAI-C): The State-Trait Anxiety Inventory for Children (STAI-C) was used to assess children’s anxiety levels. STAI-C is a validated and reliable self-report measure designed to evaluate anxiety levels in children. The internal consistency of STAIC is estimated to range between 0.78 and 0.87 [23,25]. In our study, the Cronbach’s alpha coefficient was 0.84, which is consistent with previously reported values in the literature and indicates high reliability of the scale.
- Patient Monitoring Form: Developed by the researcher to record participants’ vital signs, including systolic and diastolic blood pressure, heart rate, body temperature, and SpO_2_.

#### 2.3.10. Data Collection Process

On the day of the endoscopy, patients were called in sequence to the preoperative room, where standard hospital procedures were applied to all children in both the intervention and control groups. After the 10-min standard waiting period, children were randomized into either the intervention or control group, and data collection was conducted as follows.

### 2.4. Intervention Group

Children in the intervention group were equipped with a virtual reality (VR) headset. Through the VR headset, they viewed space and underwater-themed visuals for 7 min (Figure 2 presents a scene from these visuals). These visuals were generated using ChatGPT (DALL·E 3) AI-based image generation tools and were optimized in terms of colors, lighting, and scene composition to create an environment conducive to reducing anxiety levels in children. The AI-generated videos were processed and refined using Kling AI (version 1.5) to enhance visual coherence and ensure smoother transitions.

Additionally, calming music was generated using SUNO AI (version V3) to support relaxation in children. In the selection process, melodies with frequency ranges that could promote relaxation and align with the VR experience were prioritized.

Immediately after the VR application, the vital signs of the children in the intervention group were measured and recorded in the Patient Monitoring Form. Following this, the State-Trait Anxiety Inventory for Children (STAIC) was administered to assess their emotional state at that moment. Including the VR exposure, the entire intervention process for each participant lasted approximately 10 min.

### 2.5. Control Group

Children in the control group did not wear a VR headset. As per hospital protocol, their vital signs were measured after the 10-min standard waiting period and recorded in the Patient Monitoring Form. Immediately after this measurement, the STAIC was administered to evaluate their emotional state at that moment. The entire process for each participant in the control group lasted approximately 3 min.

#### Data Analysis

A multivariate analysis of variance (MANOVA) was conducted to test whether multiple dependent variables interacted and varied between the intervention and control groups. A linear relationship was found among each independent variable, and no multicollinearity issues were detected. The dataset was examined for outliers using the Mahalanobis distance. The critical value for six continuous dependent variables (STAIC, systolic and diastolic blood pressure, heart rate, temperature, and SpO_2_) is 22.46, and the values in the study were below this threshold (Mahal. Distance = 19.34). Since the Box’s Test of Equality of Covariance Matrices showed a significance value less than 0.05, Pillai’s Trace values were used in the results tables. Levene’s Test of Equality of Error Variance results were greater than 0.05.

Descriptive statistics such as frequency, percentage, mean, standard deviation, minimum, and maximum values were presented. Analyses were performed with a 95% confidence interval, and a significance level of *p* < 0.05 was considered. The effect size was assessed using the eta-squared (η^2^) value. An η^2^ value between 0.01 and 0.06 was considered small, between 0.06 and 0.14 moderate, and greater than 0.14 large. Statistical analyses were conducted using SPSS version 26.

## 3. Results

When Table 1 is examined, no significant differences were found between the experimental (*n* = 40) and control (*n* = 40) groups in terms of gender (68.8% female, 31.3% male; *p* > 0.05), age (26.2% aged 8–12 years, 73.8% aged 13–17 years; *p* > 0.05), educational level (66.3% high school, 25% middle school, 8.8% primary school; *p* > 0.05), chronic disease status (91.3% no chronic disease, 8.8% with chronic disease; *p* > 0.05), and previous endoscopy experience (86.3% no previous endoscopy, 13.8% with previous endoscopy; *p* > 0.05). However, a significant difference was found between the groups in terms of the reason for endoscopy (*p* < 0.05). This difference is explained by the fact that there were more participants presenting with intestinal complaints in the control group (*n* = 7) compared to the experimental group (*n* = 2) and more participants presenting with stomach complaints in the experimental group (*n* = 20) compared to the control group (*n* = 8). Apart from this, it was concluded that the intervention applied to the experimental group was independent of the demographic characteristics of the participants (Table 2).

When the means of the dependent variables for the experimental and control groups were compared, it was found that the experimental group had lower values for STAIC (36.03 ± 1.9), systolic blood pressure (89 ± 7), diastolic blood pressure (61 ± 5), heart rate (89 ± 10), and temperature (36.56 ± 0.33) compared to the control group. However, the SpO_2_ value (98 ± 1) in the experimental group was higher than that of the control group.

According to Table 3, in terms of effect sizes, the intervention had a large effect size on reducing STAIC scores (η^2^ = 0.802), improving SpO_2_ levels (η^2^ = 0.392), and lowering systolic blood pressure (η^2^ = 0.158) in the experimental group compared to the control group. It was observed that the intervention had a medium effect size on diastolic blood pressure (η^2^ = 0.060) and a small effect size on heart rate (η^2^ = 0.050) in the experimental group.

## 4. Discussion

This study provides new insights into the effective use of AI-assisted virtual reality (VR) applications for managing pre-procedural anxiety in pediatric patients undergoing endoscopy. Previous research has demonstrated the effectiveness of VR as a non-pharmacological tool for reducing preoperative anxiety [26,27,28]. However, this study specifically examines the potential of AI-generated, personalized VR content in clinical applications. Our findings indicate that children in the intervention group exhibited significantly lower anxiety levels compared to the control group, supporting the role of AI-assisted VR as an effective, non-pharmacological anxiety management tool.

Additionally, this study highlights the significance of personalization in VR content and emphasizes AI’s ability to generate individualized experiences tailored to children’s interests and developmental levels. The AI-assisted VR content used in this study aligns with previous research findings on VR-based interventions that did not incorporate AI-generated materials [29,30,31,32]. Furthermore, the flexibility and efficiency of AI in content generation enhance the accessibility and applicability of VR interventions in clinical settings. These findings strengthen the evidence supporting the integration of AI-driven VR applications in pediatric medical care and underscore the need for further large-scale investigations.

In the literature, numerous studies demonstrate that VR reduces pain, stress, and anxiety in children [4,13,33]. In this study, the positive effect of the VR application on systolic and diastolic blood pressure, consistent with the findings of other studies, indicates that this method is effective in regulating the physiological stress response. In our study, a significant reduction in systolic blood pressure was observed during and after the procedure in the VR group, supporting the improvement in cardiovascular parameters due to decreased sympathetic nervous system activity through the distraction method. Similarly, in a study conducted by Gökçe et al. (2023) it was reported that VR applications reduced systolic blood pressure and were effective in stress management [34]. Turan et al. (2024) also reported a significant reduction in systolic and diastolic blood pressure following a VR intervention, with no such changes observed in the control group [35]. Both studies emphasized that the distraction effect of VR is an important tool for regulating stress-induced hemodynamic changes. These findings support the use of VR applications, especially in clinical situations that lead to high stress levels, such as invasive procedures.

In patients experiencing anxiety, irregularities and changes in respiration are often the first observed symptoms during examinations. Additionally, various physiological reactions such as muscle tension, cold sweating, trembling, dizziness, and changes in blood pressure and heart rate are frequently observed [36,37]. One of the most observed initial signs in anxious patients during examinations is respiratory irregularity and changes. Moreover, physiological reactions such as muscle tension, cold sweating, trembling, dizziness, and changes in blood pressure and heart rate are also frequently noted. In our study, it was observed that children in the VR group had lower heart rates (η^2^ = 0.05) and higher SpO_2_ levels (η^2^ = 0.392). These findings suggest that the VR application may be associated with heart rate and SpO_2_ levels Similarly, Turan et al. (2024) reported significant improvements in heart rate, respiratory rate, and SpO_2_ values in the intervention group following the VR application, whereas no such changes were found in the control group [35]. Furthermore, in a randomized controlled study by Sweta et al. (2019) involving local anesthesia, it was reported that the VR application reduced heart rate and increased peripheral oxygen saturation (SpO_2_) [38]. Another study conducted on patients undergoing port catheter implantation demonstrated that VR goggles positively impacted systolic and diastolic blood pressure, heart rate, and respiratory rate by reducing these parameters and increasing SpO_2_ [39]. When these findings are collectively evaluated, it is understood that the VR application suppresses sympathetic nervous system activity, ensuring hemodynamic and respiratory stability.

All findings indicate that the VR application created using artificial intelligence technology is an effective non-pharmacological method for managing anxiety and physiological stress in children. In our study, the VR application, featuring AI-designed space and underwater-themed videos, was found to have a large effect size on reducing STAIC scores (η^2^ = 0.802), improving SpO_2_ levels (η^2^ = 0.392), and lowering systolic blood pressure in the experimental group compared to the control group. It also had a medium effect size on diastolic blood pressure (η^2^ = 0.060) and a small effect size on heart rate (η^2^ = 0.05). These results demonstrate that VR suppresses sympathetic nervous system activity through distraction, thereby producing positive effects on both psychological and physiological parameters. Our findings align with previous studies reporting the positive effects of VR on STAIC scores, heart rate, SpO_2_, and blood pressure [35,38,39]. These results support that AI-assisted VR applications are an effective tool for managing anxiety and physiological stress during invasive procedures and that their widespread use in clinical practice could positively contribute to health outcomes.

A key contribution of this study is the provision of new evidence supporting the effectiveness of AI-assisted VR applications in managing pre-endoscopic anxiety in children. While previous research has demonstrated the efficacy of VR in reducing pediatric anxiety, this study specifically examines how AI-driven content generation can enhance VR’s impact. With the increasing efficiency and cost-effectiveness of AI-powered content creation, the development of personalized VR experiences tailored to each child’s unique interests may become more accessible and scalable in clinical settings.

Nevertheless, further research is required to comprehensively assess the relationship between the degree of VR content personalization and its effectiveness in reducing anxiety. Future studies should explore the influence of age groups, clinical conditions, and thematic variations in VR content on pediatric anxiety management to refine its clinical applicability.

## 5. Limitations

This study has several strengths. First, the use of AI-assisted virtual reality (VR) technology provides an innovative, cost-effective, and scalable approach to reducing pre-procedural anxiety in pediatric patients. Second, conducting the study in a real clinical setting enhances the applicability of the findings to routine medical practice. Third, the inclusion of a well-defined control group allows for a more accurate assessment of the intervention’s effectiveness. Additionally, the use of the State-Trait Anxiety Inventory for Children (STAIC), a validated and widely used anxiety assessment tool, strengthens the reliability of the study results.

However, this study also has some limitations. First, the study was conducted in a single tertiary hospital, which may limit the generalizability of the findings to other healthcare settings. Second, the intervention was limited to a passive VR experience, and future research could explore the potential benefits of interactive or gamified VR applications. Third, while the sample size was sufficient to detect statistically significant differences, conducting studies with larger sample sizes across multiple centers could further enhance the reliability and applicability of the findings. Fourth, baseline vital signs were not recorded prior to the intervention. The randomization process helps minimize potential baseline differences between groups. However, future studies should consider collecting baseline physiological data to further strengthen methodological rigor and allow for a more comprehensive analysis of the intervention’s effects.

Future studies should consider multi-center designs, comparisons of different VR approaches, and long-term follow-ups to evaluate the sustainability and effectiveness of the intervention.

## 6. Conclusions

This study demonstrated that the VR application designed with artificial intelligence technology is an effective non-pharmacological method for reducing anxiety and managing physiological stress in children prior to invasive procedures such as endoscopy. The findings revealed that VR reduces heart rate, increases peripheral oxygen saturation, and significantly lowers both systolic and diastolic blood pressure. Specifically, in high-stress procedures like endoscopy, AI-supported VR applications were suggested to suppress sympathetic nervous system activity, thereby supporting hemodynamic stability. The effectiveness of AI-assisted VR applications in reducing stress and anxiety in children during endoscopy highlights their significant potential for minimizing the adverse effects of such procedures. Therefore, the widespread use of VR in clinical practice could be considered a strong alternative, particularly for reducing pre-endoscopy anxiety in the pediatric population and supporting positive health outcomes. Future studies may aim to comprehensively evaluate the effectiveness of AI-assisted VR applications in different clinical settings and larger sample groups.

## Figures and Tables

**Table 1:** Descriptive Statistics of Independent Variables.

	Experimental (*n* = 40)		Control (*n* = 40)		Total (*n* = 80)			
	C	%	C	%	C	%	Test Statistic	Sig.
Gender								
Female	27	67.5	28	70	55	68.8	χ^2^ = 0.058	0.809
Male	13	32.5	12	30	25	31.3		
Age								
8	4	10	0	0	4	5	ET = 11.067	0.271
9	1	2.5	1	2.5	2	2.5		
10	1	2.5	0	0	1	1.3		
11	2	5	4	10	6	7.5		
12	6	15	2	5	8	10		
13	1	2.5	4	10	5	6.3		
14	6	15	4	10	10	12.5		
15	4	10	4	10	8	10		
16	9	22.5	11	27.5	20	25		
17	6	15	10	25	16	20		
Educational Level								
Primary	6	15	1	2.5	7	8.8	ET = 3.941	0.139
Secondary	9	22.5	11	27.5	20	25		
High School	25	62.5	28	70	53	66.3		
Chronic Disease								
Yes	6	15	1	2.5	7	8.8	ET = 2.5	0.108
No	34	85	39	97.5	73	91.3		
Reason for Endoscopy **								
Intestinal	7	17.5	2	5	9	11.3	ET = 10.8	0.01 *
Esophagus	3	7.5	0	0	3	3.8		
Stomach	8	20	20	50	28	35		
General	22	55	18	45	40	50		
Previous Endoscopy								
Yes	5	12.5	6	15	11	13.8	ET = 0.105	0.745
No	35	87.5	34	85	69	86.3		

C: Count; %: Column N %; χ^2^ = Ki-Square, ET: Fisher’s Exact Test; * *p* < 0.05 ** Intestinal (Celiac, Diarrhea, Constipation); Esophagus (Esophagitis, Dysphagia); Stomach (Stomach pain, Nausea, Stomach bleeding, Stomach perforation, Heartburn); General (Dyspepsia, Hemoptysis, Anorexia, Bleeding focus, Vomiting).

**Table 2:** Descriptive Statistics of Dependent Variables.

	Experimental (*n* = 40)		Control (*n* = 40)		Total (*n* = 80)	
Dependent Variables *	$\bar{x}$ ± sd.	Min–Max	$\bar{x}$ ± sd.	Min–Max	$\bar{x}$ ± sd.	Min–Max
Inventory/Scale						
STAIC	36.03 ± 1.9	32–39	45.4 ± 2.74	38–49	40.71 ± 5.27	32–49
Vital Signs						
Systolic Blood Pressure	89 ± 7	80–100	97 ± 10	80–129	93 ± 10	80–129
Diastolic Blood Pressure	61 ± 5	50–70	64 ± 6	60–83	62 ± 6	50–83
Heart Rate	89 ± 10	68–107	94 ± 15	69–130	92 ± 13	68–130
Temperature	36.56 ± 0.33	36–37.2	36.9 ± 0.44	36–37.8	36.73 ± 0.42	36–37.8
SpO_2_	98 ± 1	96–99	96 ± 1	93–98	97 ± 1	93–99

* Skewness and kurtosis ±2, $\bar{x}$: mean, sd: standard deviation, Min–Max: minimum–maximum value, STAIC: State-Trait Anxiety Inventory for Children.

**Table 3:** MANOVA Test Results for Dependent Variables by Groups.

Dependent Variable	Groups	$\bar{x}$	sd.	95% Confidence Interval		Mean Difference	F	Sig.	η^2^
				Lower Bound	Upper Bound				
STAIC	Experiment	36.0	1.9	35.3	36.8	−9.375 *	315.5	0.000	0.802
	Control	45.4	2.7	44.7	46.1	9.375 *			
Systolic Blood Pressure	Experiment	89.0	6.7	86.2	91.8	−7.525 *	14.6	0.000	0.158
	Control	96.5	10.5	93.8	99.3	7.525 *			
Diastolic Blood Pressure	Experiment	60.8	4.7	59.0	62.5	−3.025 *	6.2	0.015	0.060
	Control	63.8	6.0	62.1	65.5	3.025 *			
Heart Rate	Experiment	88.7	10.1	84.7	92.7	−5.750 *	4.1	0.046	0.05
	Control	94.5	14.8	90.5	98.4	5.750 *			
SpO_2_	Experiment	98.0	1.0	97.6	98.4	1.800 *	50.3	0.000	0.392
	Control	96.2	1.3	95.8	96.6	−1.800 *			

F = 57.878 (Pillai’s Trace), *p* = 0.000, partial eta squared = 0.826. * The mean difference is significant at the 0.05 level. Adjustment for multiple comparisons: Bonferroni; F: Pillai’s trace value. The F tests the effect of groups. This test is based on the linearly independent pairwise comparisons among the estimated marginal means. STAIC: State-Trait Anxiety Inventory for Children., η^2^ = partial eta squared.

## Data Availability

The data used in this study are available from the corresponding author upon reasonable request.
